# Hyposensitivity of C-fiber Afferents at the Distal Extremities as an Indicator of Early Stages Diabetic Bladder Dysfunction in Type 2 Diabetic Women

**DOI:** 10.1371/journal.pone.0086463

**Published:** 2014-01-23

**Authors:** Wei-Chia Lee, Han-Ching Wu, Kuo-How Huang, Huey-Peir Wu, Hong-Jeng Yu, Chia-Ching Wu

**Affiliations:** 1 Division of Urology, Kaohsiung Chang Gung Memorial Hospital and Chang Gung University College of Medicine, Kaohsiung, Taiwan; 2 Department of Urology, Dalin Tzu Chi Hospital, Buddhist Tzu Chi Medical Foundation, Chiayi, Taiwan; 3 Department of Urology, National Taiwan University Hospital, College of Medicine, National Taiwan University, Taipei, Taiwan; 4 Department of Internal Medicine, National Taiwan University Hospital, College of Medicine, National Taiwan University, Taipei, Taiwan; 5 Graduate Institution of Clinical Medicine, College of Medicine, National Taiwan University, Taipei, Taiwan; 6 Department of International Business, College of Commerce and Management, Cheng Shiu University, Kaohsiung, Taiwan; University of Pittsburgh School of Medicine, United States of America

## Abstract

**Purpose:**

To investigate the relationship between distal symmetric peripheral neuropathy and early stages of autonomic bladder dysfunction in type 2 diabetic women.

**Materials and Methods:**

A total of 137 diabetic women with minimal coexisting confounders of voiding dysfunction followed at a diabetes clinic were subject to the following evaluations: current perception threshold (CPT) tests on myelinated and unmyelinated nerves at the big toe for peroneal nerve and middle finger for median nerve, uroflowmetry, post-void residual urine volume, and overactive bladder (OAB) symptom score questionnaire. Patients presenting with voiding difficulty also underwent urodynamic studies and intravesical CPT tests.

**Results:**

Based on the OAB symptom score and urodynamic studies, 19% of diabetic women had the OAB syndrome while 24.8% had unrecognized urodynamic bladder dysfunction (UBD). The OAB group had a significantly greater mean 5 Hz CPT test value at the big toe by comparison to those without OAB. When compared to diabetic women without UBD, those with UBD showed greater mean 5 Hz CPT test values at the middle finger and big toe. The diabetic women categorized as C-fiber hyposensitivity at the middle finger or big toe by using CPT test also had higher odds ratios of UBD. Among diabetic women with UBD, the 5 Hz CPT test values at the big toe and middle finger were significantly associated with intravesical 5 Hz CPT test values.

**Conclusions:**

Using electrophysiological evidence, our study revealed that hyposensitivity of unmyelinated C fiber afferents at the distal extremities is an indicator of early stages diabetic bladder dysfunction in type 2 diabetic women. The C fiber dysfunction at the distal extremities seems concurrent with vesical C-fiber neuropathy and may be a sentinel for developing early diabetic bladder dysfunction among female patients.

## Introduction

Diabetic bladder dysfunction (DBD) occurs commonly among type 2 diabetic patients, with a reported prevalence between 25% and 87% [1]. During the early (compensated) stages, DBD is usually insidious and inconspicuous. By the time urologists are consulted, DBD has usually reached a late (decompensated) stage with a classic triad of symptoms: reduced bladder sensation, large bladder capacity, and urinary retention. Specifically, the key feature distinguishing decompensated from compensated DBD is urinary retention brought on by bladder overdistention [2]. Screening of uroflowmetry and PVR has revealed that 22% of type 2 diabetic women in a clinical setting have unrecognized DBD [3]. The pathophysiology of DBD may be attributed to diabetic polyneuropathy, detrusor myopathy and/or urothelial dysfunction. This multifactorial etiology of DBD leads to a mixed clinical picture of early stages of this disease [4]. The urodynamic findings of early stages DBD in type 2 diabetic women range from detrusor overactivity (DO) to detrusor underactivity (DU), or bladder outlet obstruction (BOO) [2].

The early stages of DBD may have a wide range of clinical manifestations and remain a weak and unclear relationship with peripheral neuropathies of distal extremities. [5], [6] The increased risk of overactive bladder (OAB) observed in diabetic patients suggests that autonomic nerve irritation, bladder oversensitivity, and DO may play important roles in the early stages of DBD. [7] Furthermore, hyposensitivity of the vesical Aδ and C fiber afferents is the major cause of DU and the progression of DBD in diabetes [1], [2], [4]. During the development of diabetic neuropathy, sensory dysfunction often precedes motor dysfunction [8]. Affected sensory nerves pass through reversible stages of hyperesthesia, which is often sub-clinical, followed by hypoesthesia, and finally anesthesia. The development of peripheral neuropathy may reflect similar impairments of the vesical afferents as well as the presence of OAB syndrome in diabetic patients. In addition, diabetic patients may have a high percentage of BOO [2], [5], [9] resulting from hyper-excitable urethral afferent neuron, and impaired relaxation of urethral smooth muscle during diabetes progression [10]. Thus, an investigation of sensory function alterations at the distal extremities of diabetic patients may provide insights into early DBD.

In an effort to elucidate the relationship between the distal symmetric diabetic neuropathy (DSDN) and early stages of autonomic DBD, we conducted a cross-sectional study of type 2 diabetic women with minimal coexisting confounders of voiding dysfunction, excluding patients with advanced DBD stage. The OAB syndrome and sensory function at the distal extremities were assessed quantitatively by using overactive bladder symptom score (OABSS) [11] and the neuroselective current perception threshold (CPT) test [12], respectively. The CPT test is capable of measuring the response of myelinated and unmyelinated fibers scattered over the distal extremities and the bladder [12], [13]. Screening of uroflowmetry and post-void residual urine volume (PVR) allowed the identification of diabetic women with voiding difficulty [3], who were subsequently subjected to urodynamic studies and the intravesical CPT test.

## Materials and Methods

### Patient Enrollment

This study was approved by the Research Ethics Committee of National Taiwan University Hospital. Before investigation the patients were informed about the procedure and they provided written informed consent. A total of 150 consecutive type 2 diabetic women who had been receiving regular follow-up at the Diabetes Outpatient Clinic of the National Taiwan University Hospital for more than 1 year and had not sought treatment for DBD from Jan 2007 to Apr 2008 were enrolled in the study. A through clinical evaluation was performed on all subjects to ensure the absence of concurrent neurological disorders. Patients with other medical conditions that could interfere with voiding function, including urinary tract infections, history of hysterectomy, previous major pelvic surgery, and evidence of vaginal prolapsed were also excluded from study. The definition and measurement of clinical outcomes were based on the guidelines provided by the American Diabetes Association [14].

### Evaluation of OAB Syndrome, Uroflowmetry, PVR and CPT Values at the Distal Extremities

The OAB syndrome was assessed by the OABSS, which evaluates symptoms of urinary frequency, nocturia, urgency, and urgent incontinence. As per the Japanese OAB guidelines, an OABSS of 3 and a minimum urgency score of 2 were required for a diagnosis of OAB [15]. All patients were screened using a free uroflowmetry test and PVR measurement via a urethral catheterization. To achieve a satisfactory flow, Liverpool nomograms were used as a reference [16]. Otherwise, a repeated measurement was performed for controlling the quality of uroflowmetry. Then, a PVR of >100 ml or maximum uroflow rate <12 ml/sec was stratified as voiding difficulty [3]. The distal extremities of CPT values were measured with a Neurometer® (Neurotron, Baltimore, MD.) [12]. The device emits graded neuroselective sine-wave current stimuli at 5, 250, and 2000 Hz for testing the response of C, Aδ, and Aβ fibers, respectively, at digitally calibrated levels from 0 to 10 mA using a unit of 0.01 mA. The gold electrode was placed on the left big toe for measuring the sensory response of the peroneal nerve and on the right middle finger for median nerve response. A single blind test with an empty emission was performed for each patient to confirm the sensory threshold and increase the reliability of this semi-objective test. Increased CPT values indicated delayed sensory response during testing. Since the Neurometer® directly excites the nerve fibers, the measures obtained are not receptor or end-organ mediated. As manufacturer’s recommendation [12], the normal mean±standard deviation of median nerve perception thresholds were as follows: 48±20 for 5 Hz, 84±31 for 250 Hz, and 237±58 for 2000 Hz. The normal mean±standard deviation of peroneal nerve perception thresholds were defined as: 78±32 for 5 Hz, 118±37 for 250 Hz, and 323±76 for 2000 Hz. The thresholds above these values for any frequency were deemed to be abnormal.

### Urodynamic Studies and Intravesical CPT Test

At present, overactive bladder syndrome is not an absolute indication for urodynamic studies, and intravesical CPT test is a relatively invasive procedure. Therefore, diabetic women stratified as having voiding difficulty were subjected to urodynamic studies and an intravesical CPT test. The urodynamic studies and intravesical CPT test were performed by a single investigator (Ms. Tong-Lan Wu). Multichannel water-fill urodynamic investigations comprising filling and voiding cystometry, urethral profiles, electromyography, and uroflowmetry were performed according to the standards set forth by the International Continence Society (ICS) [17]. The intravesical CPT test was used to evaluate the bladder sensory function by using a Neurometer® and a refitted 9Fr conductance catheter (MMS 5349, Medical Measurement system, Ennschede, the Netherlands) as our previous report [2]. The response of bladder C and Aδ fiber afferents on the bladder mucosa were tested at 5 and 250 Hz frequencies.

### Statistical Analysis

The mean values of the continuous variables were compared using an independent two-sample *t* test. The Fisher’s exact test was performed to compare the odds ratios between the categorized variables and groups. The associations between CPT values of distal extremities and intravesical CPT values were examined using Pearson’s correlation analyses. A *p* value <0.05 was considered statistically significant.

## Results

A total of 137 diabetic women participated in the program had complete data, and were included in the current analysis. The demographic data are shown in table 1. Based on the OABSS, 26 (19%) diabetic women were classified as having OAB syndrome. By comparison to their non-OAB syndrome counterparts, diabetic women with OAB syndrome had a significantly greater mean CPT value of the big toe at a frequency of 5 Hz (table 2).

**Table 1:** Demographic data.

No. of patients	n = 137
Mean patients age±SD	62.5±10.3
Mean No. Parity±SD	1.75±0.7
Mean kg/m^2^ body mass index	24.0±3.9
No. menopause (%)	114 (83.2)
Mean years diabetes±SD	11.1±8.1
Distribution of hemoglobulin A_1c_, n (%)	
hemoglobulin A_1c_ <7	54 (39.4)
7<hemoglobulin A_1c_ <8	38 (27.7)
8<hemoglobulin A_1c_	45 (32.8)
No. insulin therapy (%)	21 (15.3)
No. retinopathy (%)	68 (49.6)
No. distal symmetric polyneuropathy (%)	45(32.8)
No. nephropathy (%)	68 (49.6)
No. hypertension (%)	64 (46.7)
No. hyperlipidemia (%)	71 (51.8)
Bladder dysfunction diagnosed by urodynamic studies, n = 34.	
Detrusor overactivity	8 (23.5)
Detrusor underactivity	21 (61.7)
Bladder outlet obstruction	5 (14.7)

**Table 2:** Comparisons of current perception threshold (CPT) values measured at the middle finger and big toe between diabetic women with and without overactive bladder (OAB) syndrome.

	Diabetic women with OAB syndrome	Diabetic women without OAB syndrome	*p* value
n (%)	26 (19%)	111 (81%)	
OAB symptom score	5.7±2.3	1.4±1.3	<0.001*
Values of CPT test at the middle finger for median nerve			
2000 Hz	310.0±17.5	285.8±7.0	0.15
250 Hz	126.5±9.9	130.4±9.1	0.82
5 Hz	88.5±12.3	69.3±3.8	0.08
Values of CPT test at the big toe for peroneal nerve			
2000 Hz	333.2±15.2	313.8±.7.4	0.25
250 Hz	208.4±20.3	182.1±8.9	0.21
5 Hz	135.3±18.8	99.3±6.8	0.029*

Data expressed as mean±standard error.

* Student t test, *p*<0.05.

A total of 39 diabetic women met the criteria for voiding difficulty and consented to undergo urodynamic studies as well as an intravesical CPT test. Based on the definitions provided by ICS, 34 of these patients presented with urodynamic bladder dysfunction (UBD), including DU (n = 21), DO (n = 8), and BOO (n = 5). DU and BOO were referenced to Schafer and ICS nomograms [18]. By comparison to their non-bladder dysfunction counterparts, diabetic women with UBD had greater mean CPT values of the middle finger and big toe at a frequency of 5 Hz (table 3). According to the normal ranges of CPT values provided by the manufacturer of Neurometer®, diabetic women who were categorized having C fiber hyposensitivity at the middle finger (OR = 16.2, 95% CI 5.58–46.8 ) or at the big toe (OR = 3.0, 95% CI 1.34–6.75) showed significantly higher odds ratios of having UBD (table 4).

**Table 3:** Comparisons of current perception threshold (CPT) values measured at the middle finger and big toe between diabetic women with and without urodynamic bladder dysfunction (UBD).

	Diabetic women with UBD	Diabetic women without UBD	*p* value
n (%)	34(24.8)	103 (75.2)	
Values of CPT test at the middle finger for median nerve			
2000 Hz	300.8±14.8	283.9±7.5	0.31
250 Hz	133.6±8.9	126.2±5.6	0.43
5 Hz	104.5±10.1	61.1±2.6	<0.001*
Values of CPT test at the big toe for peroneal nerve			
2000 Hz	307.6±10.0	320.1±8.3	0.34
250 Hz	196.9±15.6	183.5±9.8	0.49
5 Hz	142.9±15.8	92.7±6.4	0.005*

Data expressed as mean±standard error or the number, with the percentage in parenthesis.

* Student t test, *p*<0.05.

**Table 4:** Odds ratios and 95% confidence intervals for the associations between hyposensitivity of different types of afferents testing on median and peroneal nerves and urodynamic bladder dysfunction (UBD) in diabetic women.

	Diabetic women with UBD, n = 34	Diabetic women without UBD, n = 103	odds ratio (95% CI)	*p* value
Hyposensitivity of median nerve categorized by current perception threshold test				
Aβ fibers	3(8.8%)	7(6.8%)	1.33(0.32–5.45)	0.69
Aδ fibers	5(14.7%)	12(11.6%)	1.31(0.42–4.02)	0.64
C fibers	17(50%)	13 (12.6%)	16.2(5.58–46.8)	<0.001*
Hyposensitivity of peroneal nerve categorized by current perception threshold test				
Aβ fibers	3(8.8%)	14(13.6%)	0.62(0.17–2.29)	0.46
Aδ fibers	14(41.2%)	37(35.9%)	1.25(0.57–2.76)	0.58
C fibers	18(52.9%)	39(37.8%)	3.00(1.34–6.75)	0.008*

Data expressed as numbers with percentages in parentheses.

* Fisher’s exact test, *p*<0.05.

**Figure 1:**
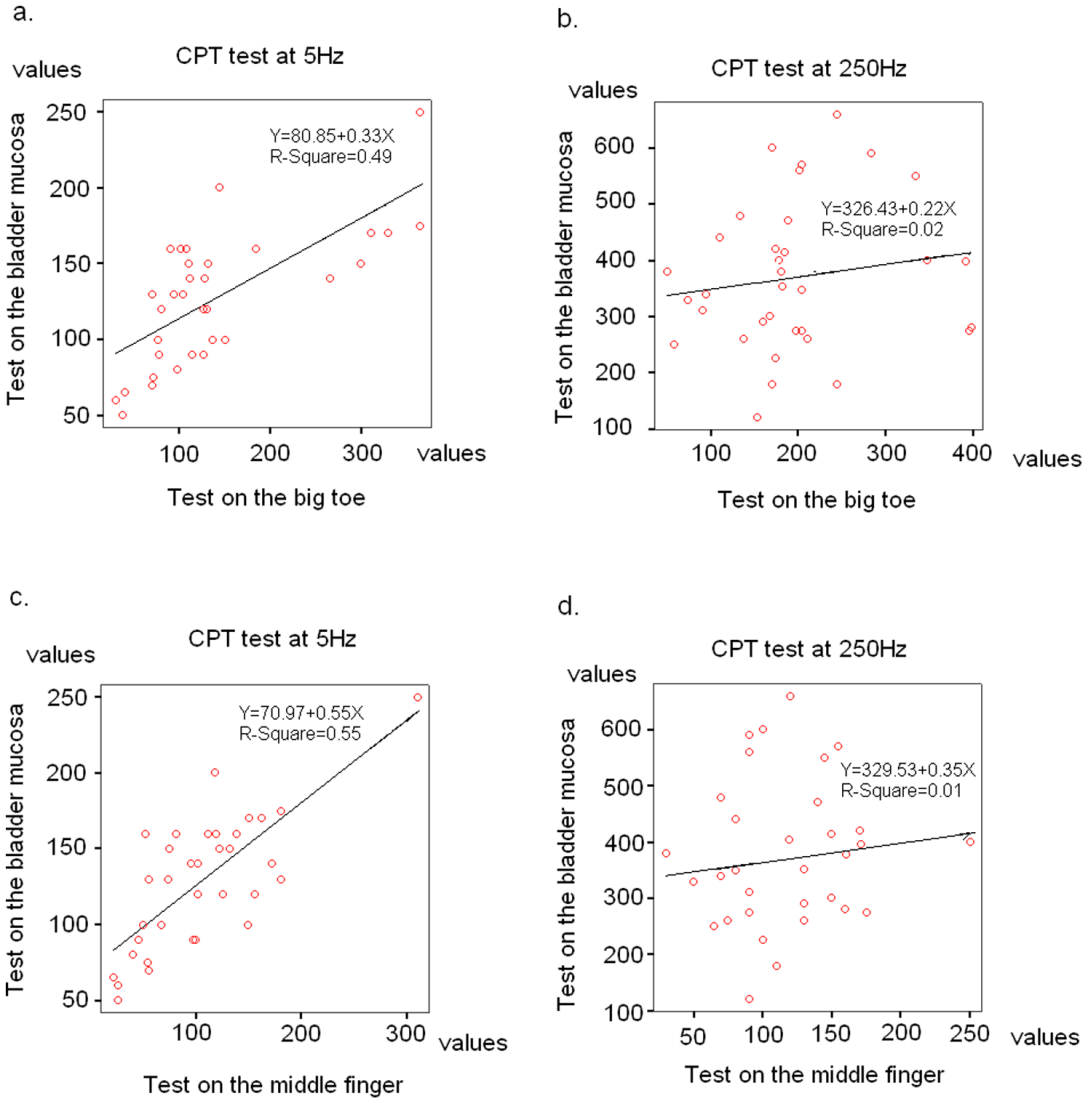
Associations of CPT values between distal extremities and bladder mucosa among diabetic women with UBD were examined by Pearson’s correlation analyses. (a) The 5 Hz CPT test values at the big toe were significantly associated with the 5 Hz CPT test values of bladder mucosa.(p<0.001) (b) No significant association between big toe and bladder mucosa at 250 Hz CPT values. (c) Significant association of 5 Hz CPT values between middle finger and bladder mucosa. (p<0.001) (d) No significant association noted at 250 Hz CPT values between middle finger and bladder mucosa.

In an effort to develop a potential screening strategy for early DBD, the association between the distal extremity CPT values and intravesical CPT values was studied in the UBD group. As illustrated in Figure 1a and c, CPT values measured at the big toe and middle finger were significantly associated with intravesical CPT values at a frequency of 5 Hz. However, the association between CPT values of the extremities and the bladder at a frequency of 250 Hz was not significant.

## Discussion

Our study revealed that 19% and 24.8% of diabetic women seen in a clinical setting had previously unrecognized OAB syndrome and UBD, respectively. Diabetic women with the OAB syndrome had a significantly greater mean 5 Hz CPT value on the big toe by comparison to values obtained from their non-OAB counterparts. This finding indicates that the C-fiber sensory function of the peroneal nerve is delayed among diabetic women with the OAB syndrome. Furthermore, by comparison to the group of diabetic women without UBD, the UBD group showed greater mean CPT values at the frequency of 5 Hz on the upper and lower extremities, indicating that the UBD group has advanced C fiber sensory neuropathy of the distal extremities. On the other hand, the diabetic women categorized as C-fiber hyposensitivity at the middle finger or big toe also had higher odds ratios of UBD. The 5 Hz CPT values for peroneal and median nerve were positively associated with the intravesical CPT values on the bladder mucosa in the UBD group. However, no significant association was observed between CPT values of distal extremities and bladder mucosa at a frequency of 250 Hz. This finding suggests that myelinated Aδ fiber function of the distal extremities does not relate to the progression of vesical autonomic neuropathy and DBD in the early stages among diabetic women. Conversely, the development of C-fiber neuropathy at distal extremities and bladder seems to be simultaneous. Hence, the hyposensitivity of unmyelinated C-fiber function at the distal extremities reflects progressive diabetic polyneuropathy, and predicts the likelihood of developing early DBD among female patients.

The presentation of urinary urgency or the OAB syndrome in diabetic patients remains a controversy. Traditionally, DBD has been described as a triad of decreased sensation, increased bladder capacity, and poor emptying. It has previously been suggested that a diabetic patient would not sense urinary urgency due to a combination of reduced sensation and increased capacity of the bladder [19]. However, several reports indicated that diabetic patients may have a high prevalence of OAB syndrome [20]–[22]. A recent large-scale investigation including 1359 diabetic patients, reported that 24.8% of male and 20.1% of female diabetic Taiwanese have the OAB syndrome [21]. In the present study, we excluded diabetic patients with an advance stage of flaccid bladder, which included 19% of female diabetic patients presenting with the OAB syndrome. Our study also indicated that diabetic women with OAB syndrome may have a poorer C-fiber sensory function in the territory of the peroneal nerve. The OAB syndrome presenting in diabetic women might be a part of manifestations of diabetic polyneuropathy with dissociated presentations. The diabetic patient presenting with OAB syndrome could be associated with bladder oversensitivity, DO, or even BOO [9], which may result from autonomic afferents and efferents disturbance of diabetes [10], [23]. During the early stages of DBD, the bladder can over-express nerve growth factor, and M2 and M3 muscarinic receptors, while dysregulating urothelial chemicals, enzyme, and receptors as a result of hyperglycemic insults or metabolic perturbations [23], [24]. Afferent noise and instability of the detrusor muscle may lead to the bladder oversensitivity and DO observed in the urodynamic studies of diabetic patients [24], [25].

The current study demonstrated that hyposensitivity of C-fiber sensory function at the distal extremities can be an indicator of early DBD among diabetic women. Indeed, the UBD group had higher thresholds of C-fiber at both distal peroneal and median nerves, indicating a greater intensity of peripheral nerve lesions in this group. The higher odds ratio of developing UBD in diabetic women categorized as C-fiber hyposensitivity at peroneal or median nerves also stand this viewpoint. Generally speaking, C-fibers are vulnerable and lost early in the progression of type 2 diabetes. The vast majority of patients with clinical diabetic neuropathy have a distal symmetrical form of the disorder, which progresses following a fiber length-dependent pattern of sensory and autonomic manifestations. The presentation and pathogenesis of DSDN is the result of progressive distal axonopathy [8]. The progression of distal symmetric sensory loss often begins at the toes, extends over the feet, and spreads above the knee level. When sensory loss extends above knee level, it also develops over the fingers and progresses further. Thus, diabetic patients with advanced neuropathy may have an increased risk of DBD [26]. Moreover, researchers reported that a greater distal extremity CPT value of diabetic patients was associated with the higher prevalence of increased fasting plasma glucose, proliferative diabetic retinopathy, macroalbuminuria, insulin treatment, and a longer duration of diabetes since diagnosis in different populations [12], [27], [28]. The hyposensitivity of C-fiber sensory function of the lower and upper extremities and UBD commonly occurred together in diabetic patients, highlighting the progression and severity of diabetic polyneuropathy. Given that C-fiber functional defects of distal extremities may precede diabetic bladder remodeling causing by overdistention and are more likely to be improved through glycemic control [1], [29], the peripheral CPT test may be particularly valuable in assessing interventions to prevent or delay the progress of vesical neuropathy. Therefore, screening of C-fiber sensation at the distal extremities with a non-invasive procedure could estimate the progression of vesical C-fiber neuropathy and identify DBD cases.

Our study revealed that alterations in unmyelinated C-fiber sensation may occur simultaneously in the bladder and distal extremities of diabetic patients. However, alterations of C-fiber function as measured by the CPT test at the distal extremities are not accurate predictors of severity and types of bladder dysfunction in diabetic individuals. Positive associations were observed in this study between unmyelinated C-fiber response at the distal extremities and the bladder mucosa. In contrast, the responses of the CPT test of the myelinated Aδ fiber at the distal extremities and bladder mucosa were not related. In the early diabetic polyneuropathy, the dissociation between the function of small-unmyelinated and large-myelinated fibers is common [8]. Small-fiber functions are severely affected at an early stage, whereas large-fiber functions are initially spared. In fact, sensory neuropathy may be completely silent and detected only by systematic neurological examinations. Previously, we have reported that the decreased sensation of Aδ and C fibers of the bladder can cause DU and reduce bladder voiding efficiency among diabetic patients [2]. Vesical C-fibers could be involved in the initiation of bladder emptying, while the hyposensitivity of vesical C-fibers may cause a large bladder capacity [30]. The hyposensitivity of vesical Aδ fibers is thought to be responsible for the bladder overdistention and development of DU in diabetes [19]. However, diabetes can lead to other types of voiding dysfunction, such as bladder oversensitivity, DO, and an inability to relax the urethra as a result of urothelial dysfunction, diabetic detrusor myopathy, or urethral nitric oxide imbalance [23], [31]. Taken together, the responses of myelinated and unmyelinated fibers of the distal extremities cannot be used to assess the precise bladder dysfunction in diabetic patients. Further urodynamic studies are needed to differentiate different types of DBD. Our study has some limitations. The current study is a hospital based cross-sectional study. A community based longitudinal study is needed further to verify the disease progression relationship between diabetic somatic peripheral neuropathy and autonomic visceral organs neuropathy.

## Conclusion

Using electrophysiological evidence the present study identified an association between hyposensitivity of C fiber function at the distal extremities and early stages DBD in diabetic women. The alterations of C fiber function at distal extremities and bladder mucosa may occur simultaneously in these patients; this was not true of Aδ fiber function. The impairment of C fiber function at the distal extremities is a sign of peripheral polyneuropathy and may be a sentinel for developing early diabetic bladder dysfunction among female patients.
